# Shade Nets Modulate Photosynthetic Balance and Antioxidant Homeostasis to Improve Fruit Quality in Ponkan

**DOI:** 10.3390/plants15182870

**Published:** 2026-09-19

**Authors:** Qiuyou Chen, Yang Yang, Xia Qiu, Min Ding, Yitong Lv, Zhimin Cheng, Siyuan Li, Ping Wang

**Affiliations:** 1 Institute of Genetics and Breeding in Horticultural Plants, College of Horticulture, Fujian Agriculture and Forestry University, Fuzhou 350002, China; 2 Key Laboratory of Ministry of Education for Genetics, Breeding and Multiple Utilization of Crops, Fujian Agriculture and Forestry University, Fuzhou 350002, China

**Keywords:** canopy microclimate, chlorophyll fluorescence, *Citrus reticulata*, leaf gas exchange, redox homeostasis, sugar–acid composition

## Abstract

To clarify the effects of shade-net covering on sunburn control and fruit quality of ponkan, white, gray, black, and blue nets with differing light transmittance were applied to assess the canopy microenvironment, leaf photosynthesis, peel antioxidant system, and fruit quality. All shading treatments significantly reduced fruit surface temperature and light intensity compared with the unshaded control. Light transmittance was significantly higher under white (26.49%) and blue (25.69%) nets than under gray (12.97%) and black (12.58%) nets. Consequently, sunburn incidence decreased significantly from 31.41% in the control to 5.47–11.13% under shading. Regarding leaf photosynthesis, white and blue nets maintained a significantly higher net photosynthetic rate (*P*_n_) and PSII photochemical efficiency than gray and black nets, whereas gray and black nets showed no significant difference in *P*_n_ from the control. All shading treatments significantly lowered reactive oxygen species (ROS) and malondialdehyde (MDA) levels, alleviating peel oxidative injury. By contrast, the sunburn peel of the unshaded control accumulated high levels of ROS and MDA and activated SOD, POD, CAT, and the AsA-GSH cycle, thereby elevating total antioxidant capacity (T-AOC). At fruit maturity, shading generally improved juice yield and nutritional quality. White and gray nets enhanced soluble solids content (SSC), white and blue nets improved the sugar–acid ratio, and gray, black, and blue nets increased vitamin C content, while the white net showed the best performance in soluble sugar accumulation. These results indicate that white and blue shade nets are more effective in optimizing the canopy microenvironment of ponkan. Shading followed by post-shading light recovery alleviates fruit sunburn and enhances fruit quality.

## 1. Introduction

Citrus, belonging to the subfamily Aurantioideae (Rutaceae), has long been among the most widely cultivated and productive fruit crops worldwide [1]. Ponkan (*Citrus reticulata* Blanco) is a commercially valuable variety valued for its regular shape, easy-peeling rind, crisp and juicy flesh, pleasant flavor, and few seeds, making it important for both the fresh market and the processing industry [2]. However, in summer, extreme high temperature and high-intensity solar radiation frequently induce fruit sunburn, leading to peel damage, dehydration, and flesh damage, which significantly reduce yield and commercial quality, and cause direct economic losses [3]. With global warming, the frequency of extreme summer heat has increased in subtropical regions, exacerbating not only fruit sunburn but also inhibition of leaf photosynthesis and disruption of cellular redox homeostasis, thereby further compromising fruit quality [4]. Therefore, effective strategies to control sunburn in ponkan are urgently needed to secure producers’ income and meet consumer demands for high-quality fruit.

Sunburn is a common physiological disorder in numerous fruit trees, including apple, grape, citrus, loquat, pomegranate and kiwifruit. Its severity differs across temperate, subtropical and tropical climatic zones [4,5]. As a major disorder reducing fruit quality and marketable yield, sunburn has become increasingly severe under the dual pressures of global warming and the adoption of dwarf, high-density orchards which exacerbate sunburn by increasing fruit exposure to direct solar radiation and altering the canopy microclimate with less natural shading. The primary cause of sunburn is the sustained elevation of fruit surface temperature induced by direct strong sunlight, which manifests as three types of injury: thermal damage, ultraviolet radiation damage, and photooxidative damage of illuminated tissues [4]. It is widely recognized that the combination of high temperature and strong light is the key condition for sunburn [6]. Fruits on the outer canopy, especially on the western and southern aspects, as well as those on southwest and west-facing slopes, receive higher light intensity and greater heat load, showing a significantly elevated sunburn risk [7]. In ponkan, the fruit expansion stage coincides with the high-temperature, high-light season, when the peel has low stress resistance. Sunburn mainly occurs from July to September, causing high damage rates in major production regions and becoming a critical constraint on productivity.

Shade nets are widely used in subtropical fruit production. They can effectively attenuate solar radiation and reduce canopy temperature, providing reliable control of fruit sunburn [8]. Studies have shown that shade net coverage lowers canopy temperature and leaf heat stress, reduces sunburn and leaf wilting, and ensures normal tree growth [9]. Shade nets protect against physical damage from strong wind and hail [10]. In particular, colored nets (e.g., white and blue) can also reduce sunburn, thereby improving orchard economic returns. Shade nets regulate the canopy microenvironment by altering light transmittance, reflectance, and spectral composition [11]; different net colors can significantly modify light quality ratios [12], thereby affecting tree physiological metabolism and photosynthetic processes [13].

Light intensity and light quality are key factors affecting photosynthesis [14]. Excessive irradiance readily causes photoinhibition, damaging PSII reaction centers and reducing photosynthetic efficiency [15,16]. Shading that reduces full-sunlight irradiance by 33–55% alleviates photoinhibition, increases chlorophyll content, optimizes photosynthetic electron transport and light energy utilization, and improves leaf photosynthetic performance [17]. However, excessive shading by shade nets decreases the net photosynthetic rate, which is unfavorable for photosynthate accumulation [14]. Depending on their spectral transmission characteristics, shade nets of different colors exert distinct regulatory effects on photosynthetic pigments (i.e., chlorophyll *a*, chlorophyll *b*, and total carotenoids), the chlorophyll *a*/*b* ratio, chlorophyll fluorescence parameters, and the photosynthetic rate [18]. Meanwhile, shade coverage affects fruit growth by modifying the microenvironment (light intensity, temperature, humidity), altering the accumulation of soluble solids, organic acids, vitamin C, and sugar (e.g., sucrose, glucose, and fructose) components, thereby influencing fruit flavor and quality [19].

Under high-temperature and high-light stress, plants accumulate excessive reactive oxygen species (ROS) [15,20], triggering membrane lipid peroxidation and malondialdehyde (MDA) accumulation, which damage the structure and function of cell membranes. Plants scavenge excess ROS through both enzymatic and non-enzymatic antioxidant systems [21]. Superoxide dismutase (SOD), peroxidase (POD), and catalase (CAT) form the core enzymatic system, while the ascorbate–glutathione (AsA-GSH) cycle serves as a major non-enzymatic antioxidant pathway [22], jointly maintaining redox homeostasis [23,24]. Shade net coverage reduces light intensity and fruit surface temperature, suppresses ROS burst, mitigates membrane lipid peroxidation [3,25], and maintains the antioxidant system at a relatively stable basal level. In comparison, sunburn stress in non-shaded fruit markedly activates the peel antioxidant system, as reflected by elevated ROS and MDA contents, increased antioxidant–enzyme activities, and higher levels of antioxidant substances [25].

To date, blue, white, black, and other shade nets have been reported for sunburn control and quality regulation in citrus, apple, grape, pitaya, and other crops [9,13,19,26]. However, systematic studies on ponkan remain limited, and the comparative effects of differently colored shade nets on the canopy microenvironment, leaf photosynthetic performance, peel antioxidant system, and fruit quality have not been fully integrated. Moreover, the relationships among microenvironmental modification, photosynthetic performance, antioxidant homeostasis, and quality formation under different net colors warrant further investigation. In this study, ponkan was used as experimental material to systematically investigate the effects of four shade nets (white, gray, black, and blue) on the orchard canopy microclimate, post-shading light recovery characteristics, leaf photosynthetic performance, peel antioxidant systems, and fruit quality. This study aims to clarify the regulatory mechanisms of shade nets with distinct spectral properties and screen optimal shading materials for alleviating ponkan fruit sunburn. The findings provide a theoretical basis and practical technical guidance for improving fruit quality in commercial ponkan cultivation.

## 2. Results

### 2.1. Effects of Different Colored Shade Nets on the Canopy Microenvironment of Ponkan

**Figure 1:**
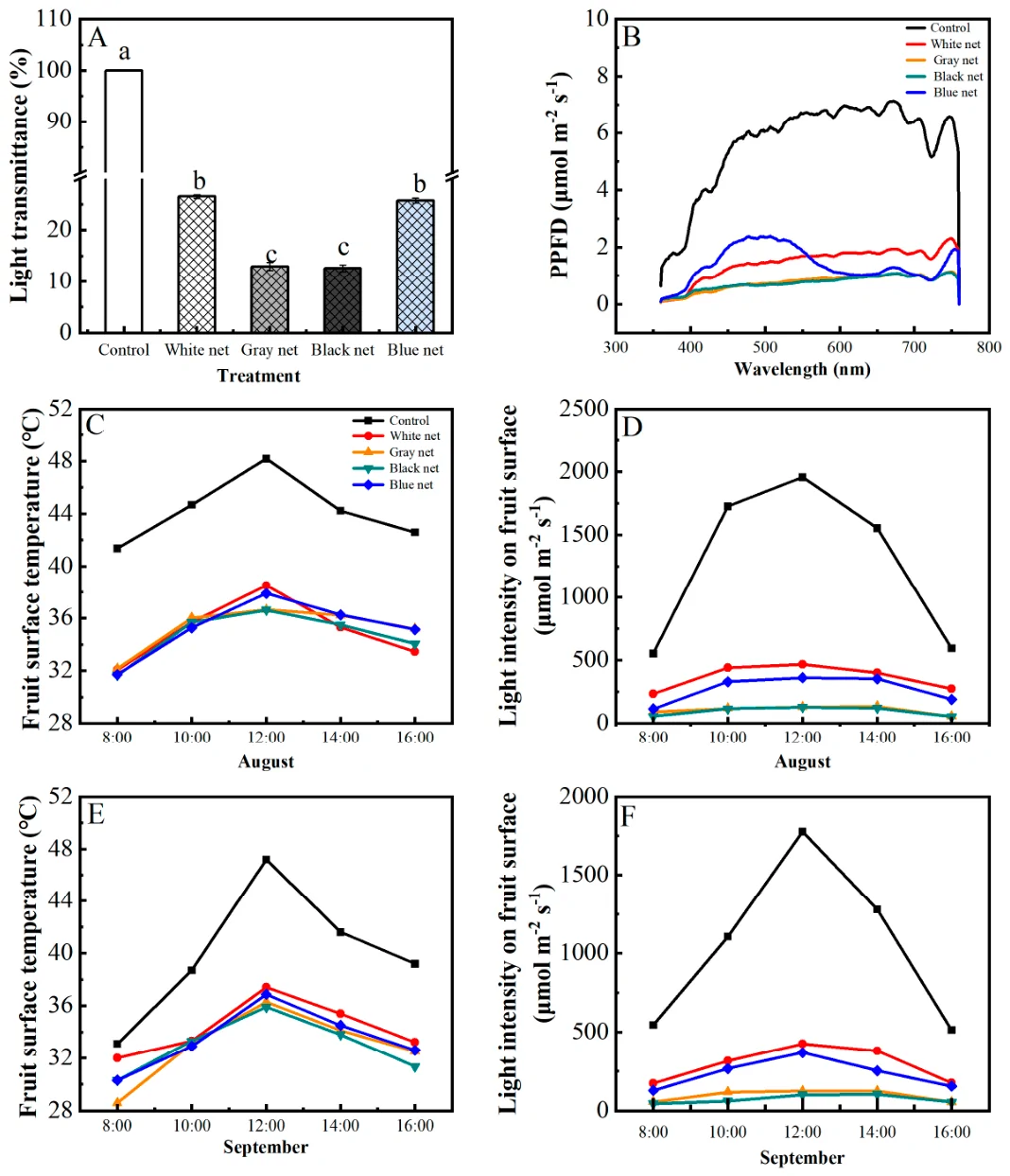
Effects of different colored shade nets on the ponkan canopy microenvironment. Light transmittance of the control and four colored shade nets (**A**), spectral distribution of PPFD (**B**), diurnal variations in fruit surface temperature in August (**C**) and September (**E**), and fruit-surface light intensity in August (**D**) and September (**F**). Statistical differences among treatments for panels (**B**–**F**) are shown in Supplementary Tables S1–S5. Different lowercase letters indicate significant differences at *p* < 0.05 by Duncan’s multiple range-test. Error bars are omitted in panels (**C**–**F**) to avoid curve overlap. Data are expressed as means ± standard error (*n* = 3).

The light transmittance of the differently colored shade nets, expressed as a percentage of full-sun control, is presented in Figure 1A. The white (26.49%) and blue (25.69%) nets were significantly higher than the gray (12.97%) and black (12.58%) nets, with no significant difference within each pair. Spectral photosynthetic photon flux density (PPFD) distribution (Figure 1B) showed that the unshaded control had the highest values across all wavelengths. The gray and black nets exhibited similar spectral curves with no significant differences across most wavelengths. The blue net showed significantly higher transmittance in the blue band (400–500 nm) and lower transmittance near 700 nm compared with other colored nets. The white net had significantly higher red-band and 740 nm transmittance than the gray and black nets, but did not differ significantly from the blue net in total transmittance. PPFD declined sharply above 760 nm in all treatments.

Colored shade nets significantly reduced fruit surface temperature and canopy light intensity versus the control (Figure 1C–F). Fruit surface temperature peaked at 12:00 in both months. In August, peak temperatures under the black (36.63 °C) and gray (37.24 °C) nets were significantly lower than under the white (38.50 °C) and blue (38.12 °C) nets, with no significant differences within each group. The same grouping pattern was observed in September (black/gray: 35.90–36.20 °C; white/blue: 37.10–37.40 °C). The unshaded control had significantly higher peak temperatures (48.18 °C in August, 47.20 °C in September) than all shading treatments.

Under our field conditions, canopy light intensity under the shading nets consistently peaked at 12:00 (Figure 1D,F). The control was significantly higher than all shaded treatments throughout the day, with midday means of 1955.00 (August) and 1777.30 μmol m^−2^ s^−1^ (September). Among shading nets, midday light intensity was significantly higher under the white net than under the blue, gray, and black nets; the blue net was significantly higher than the gray and black nets; and no significant difference was detected between the gray and black nets. This pattern was consistent across both months.

### 2.2. Effects of Different Colored Shade Nets on Photosynthetic Light Response Characteristics of Ponkan Leaves

**Figure 2:**
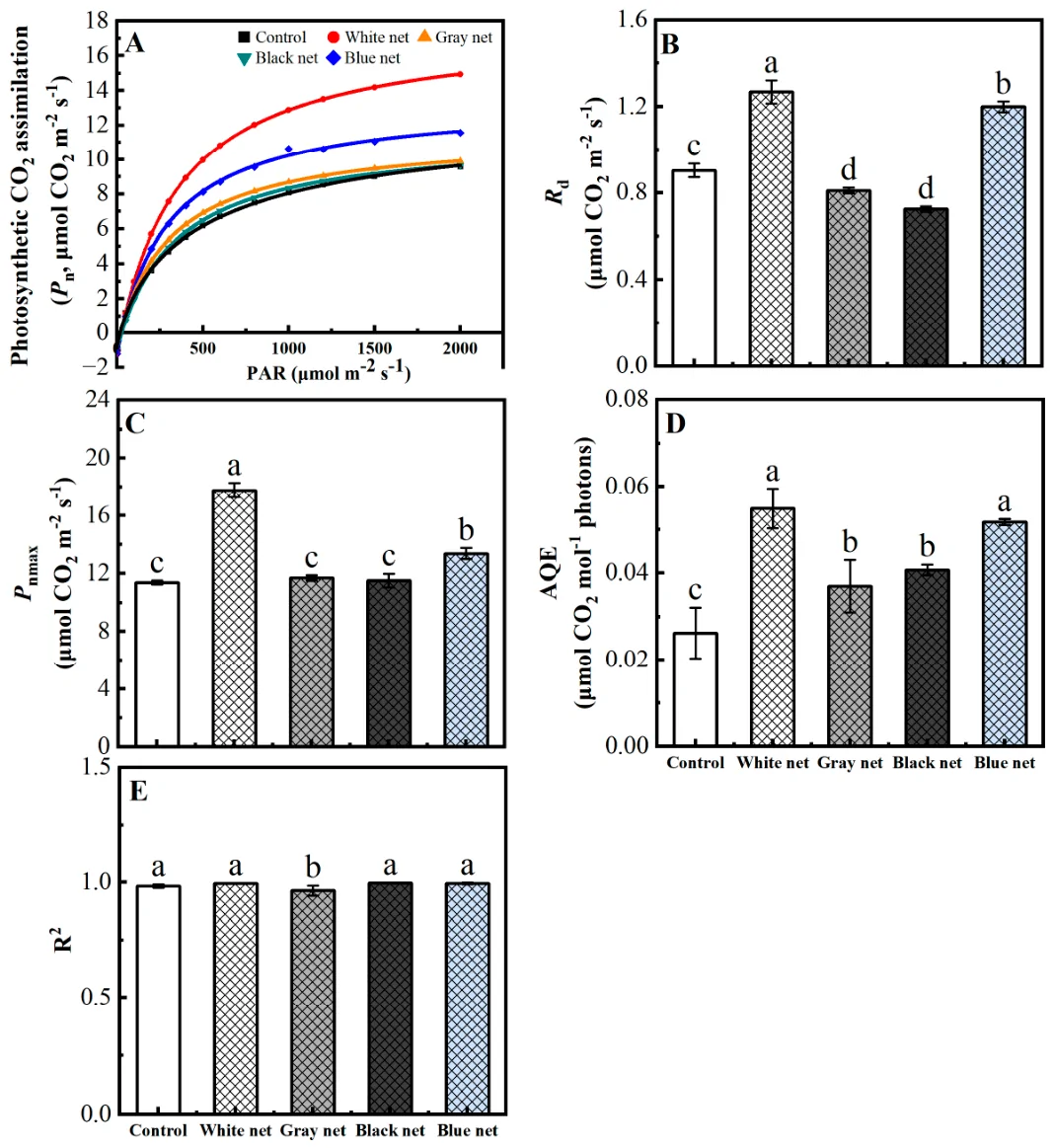
Effects of different colored shade nets on PAR-*P*_n_ light response curves of ponkan leaves. PAR-*P*_n_ response curves (**A**), fitting parameters of dark respiration rate (*R*_d_) (**B**), maximum net photosynthetic rate (*P*_nmax_) (**C**), apparent quantum yield (AQE) (**D**), and the coefficient of determination (R^2^) (**E**). Different lowercase letters above bars within the same month indicate significant differences among treatments at *p* < 0.05 by Duncan’s multiple range-test. Data are expressed as means ± standard error (*n* = 3).

The response patterns of photosynthetic CO_2_ assimilation (*P*_n_) to PAR in ponkan leaves were basically consistent across all treatment groups (Figure 2A). When PAR was below 600 μmol m^−2^ s^−1^, the leaf *P*_n_ increased rapidly; when PAR exceeded 1200 μmol m^−2^ s^−1^, the *P*_n_ tended to level off and reached a saturated state. No significant differences were detected at μmol m^−2^ s^−1^, whereas significant differences among treatments appeared at PAR > 500 μmol m^−2^ s^−1^. *P*_n_ under the white and blue nets was significantly higher than that under the control, whereas no significant difference was detected between the gray and black nets and the control. The white and blue nets also resulted in significantly higher *P*_n_ than the gray and black nets across most PAR levels. Under low-light conditions (PAR < 400 μmol μmol m^−2^ s^−1^), *P*_n_ increased more rapidly under the white and blue nets than under the other treatments, indicating superior low-light utilization capacity. The photosynthetic light response curves of leaves in all treatments were well fitted, with the coefficients of determination (R^2^) all greater than 0.96 (Figure 2B–E). For the dark respiration rate (*R*_d_) and maximum net photosynthetic rate (*P*_nmax_), the white and blue nets showed significantly higher values than the gray, black, and control treatments. The white net also had significantly higher values than the blue net, whereas no significant difference was observed between the gray and black nets. All shading treatments exhibited significantly higher apparent quantum yield (AQE) values than the full-sun control. Furthermore, the white- and blue-net treatments showed significantly higher AQE values compared with the gray and black nets, with no significant difference within each pair.

### 2.3. Effects of Different Colored Shade Nets on Photosynthetic Pigments and Chlorophyll Fluorescence Parameters in Ponkan Leaves

**Figure 3:**
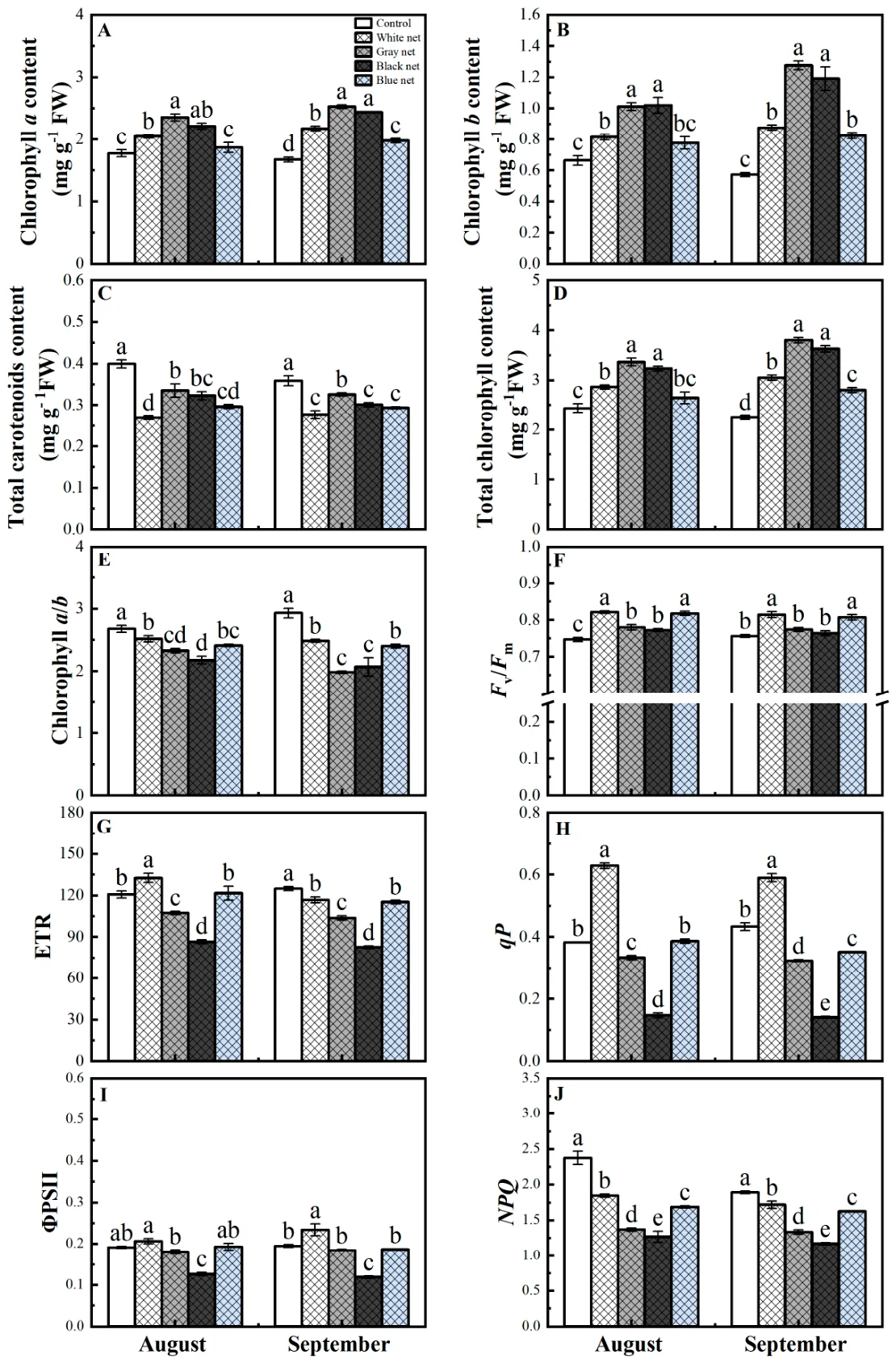
Effects of different colored shade nets on photosynthetic pigments and chlorophyll fluorescence parameters of ponkan leaves in August and September. Chlorophyll *a* content (**A**), chlorophyll *b* content (**B**), total carotenoid content (**C**), total chlorophyll content (**D**), chlorophyll *a*/*b* ratio (**E**), maximum photochemical efficiency of photosystem II (*F*_v_/*F*_m_) (**F**), electron transport rate (ETR) (**G**), photochemical quenching coefficient (*q*P) (**H**), actual photochemical efficiency of PSII (ΦPSII) (**I**), and non-photochemical quenching (*NPQ*) (**J**). Different lowercase letters above bars within the same month indicate significant differences among treatments at *p* < 0.05 by Duncan’s multiple range-test. Data are expressed as means ± standard error (*n* = 3).

In August, chlorophyll *b* and total chlorophyll concentrations in leaves of plants under the white-net, gray-net, and black-net treatments were significantly higher than those in control plants, whereas plants under the blue-net treatment showed no significant difference from the control. Among the shading treatments, chlorophyll *b* and total chlorophyll concentrations were highest in plants under the white-net treatment. By September, all shade-net treatments, including the blue-net treatment, significantly enhanced both chlorophyll *b* and total chlorophyll concentrations compared with the control. The relative increase in chlorophyll *b* was consistently larger than that in chlorophyll *a* in both months, with greater increments observed in September. The relative increases in chlorophyll *a* ranged from 5.46% to 32.85% in August and from 18.28% to 51.19% in September, while those of chlorophyll *b* ranged from 17.05% to 53.77% in August and from 44.37% to 123.61% in September. All shading treatments significantly reduced total carotenoid content compared with the control in both months (Figure 3A–D). In August, the chlorophyll *a*/*b* ratio in leaves of plants under all shading treatments was significantly lower than that in control plants under full sunlight. Among the shading treatments, the white-net treatment resulted in a significantly higher ratio than the gray-net and black-net treatments, while the blue-net treatment showed no significant difference from either the white-net or gray-net treatment (Figure 3E).

Chlorophyll fluorescence parameters are shown in Figure 3F–J. On both measurement dates (1 August and 1 September), maximum photochemical efficiency of photosystem II (*F*_v_/*F*_m_) under the white and blue nets was significantly higher than under the control, gray net, and black net. In August, *F*_v_/*F*_m_ under the gray-net and black-net treatments was significantly higher than that under the control, whereas in September, no significant difference was observed among the control, gray-net, and black-net treatments (Figure 3F). The electron transport rate (ETR) exhibited divergent monthly responses. In August, the ETR in leaves of plants under the white-net treatment was significantly higher than that in plants under all other treatments, while the ETR in plants under the blue-net treatment and control plants did not differ significantly from each other but was significantly higher than that in plants under the gray-net and black-net treatments. In September, control plants exhibited the highest ETR, significantly higher than that in plants under all shading treatments; among the shading treatments, the ETR in plants under the white-net and blue-net treatments was significantly higher than that in plants under the gray-net and black-net treatments. Notably, plants under the black-net treatment showed the lowest ETR values in both August and September. The photochemical quenching coefficient (*q*P) and actual photochemical efficiency of PSII (ΦPSII) shared identical variation trends. Across both months, *q*P was significantly higher under the white net than under all other treatments, whereas the gray and black nets showed significantly lower *q*P relative to the control. The blue net and the control did not differ significantly in *q*P in August; however, the control exhibited significantly higher *q*P than the blue net in September. For ΦPSII, plants under the black-net treatment exhibited significantly lower values than control plants in both August and September, while plants under the white-net treatment showed significantly higher ΦPSII than plants under all other treatments only in September. In September, the black net recorded the lowest ΦPSII, differing significantly from all other treatments (Figure 3G–I). Non-photochemical quenching (*NPQ*) represents thermal dissipation of excess light energy. *NPQ* under the control was significantly higher than under all shading treatments. Among the shading treatments, *NPQ* in leaves of plants under the white-net treatment was significantly higher than that in plants under the blue-net, gray-net, and black-net treatments; *NPQ* in plants under the blue-net treatment was significantly higher than that in plants under the gray-net and black-net treatments; and *NPQ* in plants under the gray-net treatment was significantly higher than that in plants under the black-net treatment (Figure 3J).

### 2.4. Effects of Different Colored Shade Nets on Leaf Gas Exchange Parameters of Ponkan

**Figure 4:**
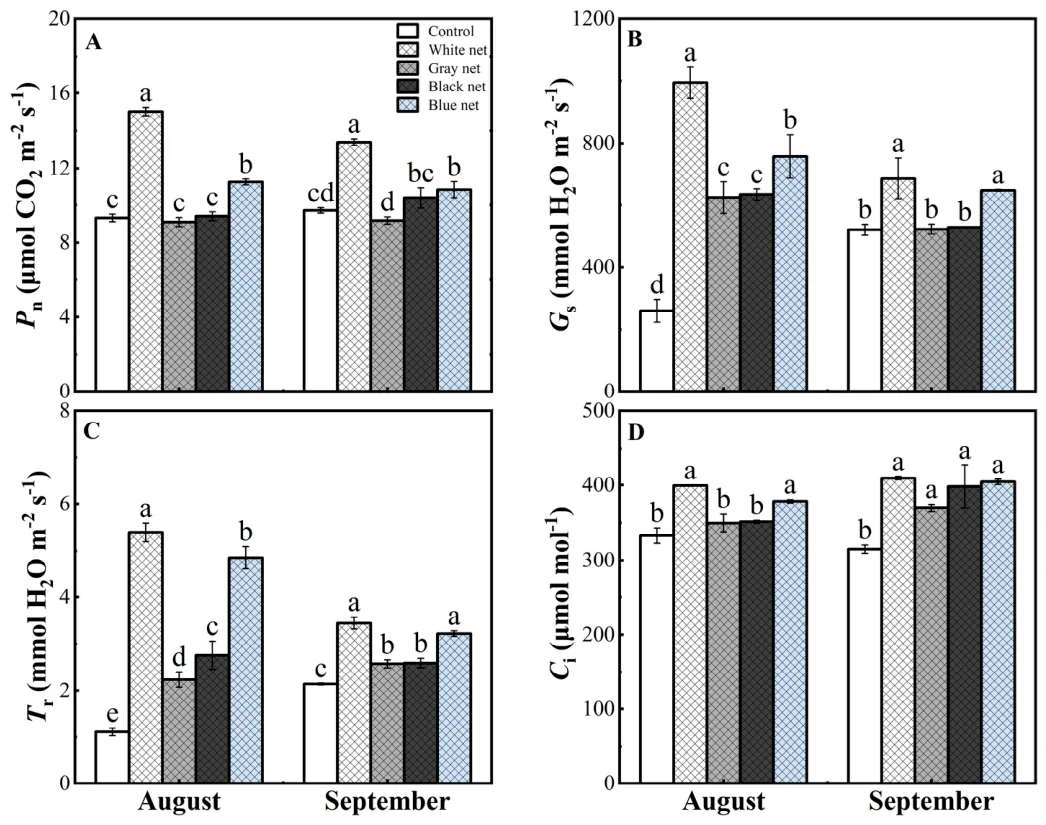
Effects of different colored shade nets on leaf gas exchange parameters of ponkan in August and September. Net photosynthetic rate (*P*_n_) (**A**), stomatal conductance (*G*_s_) (**B**), transpiration rate (*T*_r_) (**C**), and intercellular CO_2_ concentration (*C*_i_) (**D**). Different lowercase letters above bars within the same month indicate significant differences among treatments at *p* < 0.05 by Duncan’s multiple range-test. Data are expressed as means ± standard error (*n* = 3).

As shown in Figure 4A, in both August and September, the net photosynthetic rate (*P*_n_) under the white and blue nets was significantly higher than that under the control, while no significant difference was detected between the control and either the gray or black nets. Among the shading treatments, the white net resulted in significantly higher *P*_n_ than the blue, gray, and black nets in both months. In August, *P*_n_ under the blue net was significantly higher than that under the gray and black nets, whereas in September, the blue net showed significantly higher *P*_n_ than the gray net but did not differ significantly from the black net.

In August, the patterns of stomatal conductance (*G*_s_) and the transpiration rate (*T*_r_) were aligned (Figure 4B,C). *G*_s_ and *T*_r_ under the white net were significantly higher than those under all other treatments; the blue net showed significantly higher values than the gray, black, and control treatments; both the gray and black nets exhibited significantly higher *G*_s_ and *T*_r_ than the control. In September, *T*_r_ under the white and blue nets were significantly higher than those under the control, gray, and black nets. For *G*_s_, no significant differences were observed among the control, gray, and black nets. For *T*_r_, both the gray and black nets showed significantly higher values than the control. In contrast, intercellular CO_2_ concentration (*C*_i_) exhibited a distinct variation pattern. In August, *C*_i_ in leaves of plants under the white-net and blue-net treatments was significantly higher than that in control plants and in plants under the gray-net and black-net treatments, with no significant difference between control plants and plants under the gray-net or black-net treatments. In September, all shading treatments significantly elevated *C*_i_ compared with the control, but no significant differences were found among the shade-net treatments (Figure 4D).

### 2.5. Effects of Different Colored Shade Nets on O_2_$\bar{\cdot }$, H_2_O_2_ and MDA Contents in Ponkan Peel

**Figure 5:**
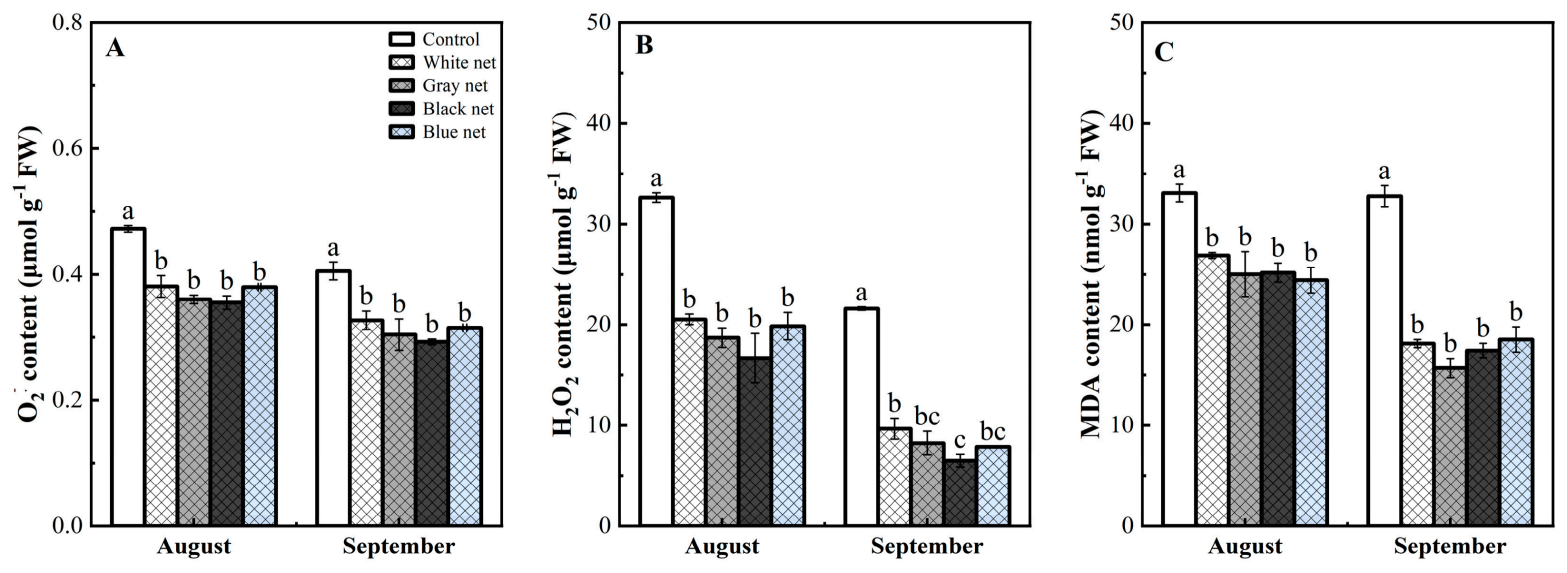
Effects of different colored shade nets on O_2_$\bar{\cdot }$, H_2_O_2_, and MDA contents in ponkan fruit peel in August and September. Superoxide anion (O_2_$\bar{\cdot }$) content (**A**), hydrogen peroxide (H_2_O_2_) content (**B**), and malondialdehyde (MDA) content (**C**). Different lowercase letters above bars within the same month indicate significant differences among treatments at *p* < 0.05 by Duncan’s multiple range-test. Data are expressed as means ± standard error (*n* = 3).

As shown in Figure 5A–C, compared with the full-sun control, all shading treatments significantly lowered superoxide anion (O_2_$\bar{\cdot }$), hydrogen peroxide (H_2_O_2_), and MDA contents in ponkan peel across both August and September, indicating alleviated membrane oxidative injury. Specifically, for O_2_$\bar{\cdot }$ levels, no significant differences were found among the four colored shade nets in either month. For H_2_O_2_ content, no significant differences were observed among the four shade-net treatments in August, which may be attributable to the relatively high standard deviation recorded for the black net despite similar changing trends across treatments. In September, statistical differences were detected among shade-net treatments: the white-net treatment possessed the highest H_2_O_2_ concentration, the gray-net and blue-net treatments presented intermediate values, whereas the black-net treatment showed the lowest H_2_O_2_ level, which was significantly lower than that under the white-net treatment. As for MDA, all four shading treatments significantly reduced its accumulation compared with the control in both months; however, no significant differences were observed among the shade nets themselves. Collectively, all four shade nets effectively suppressed ROS and MDA accumulation in ponkan peel.

### 2.6. Effects of Different Colored Shade Nets on the Antioxidant System in Ponkan Peel

**Figure 6:**
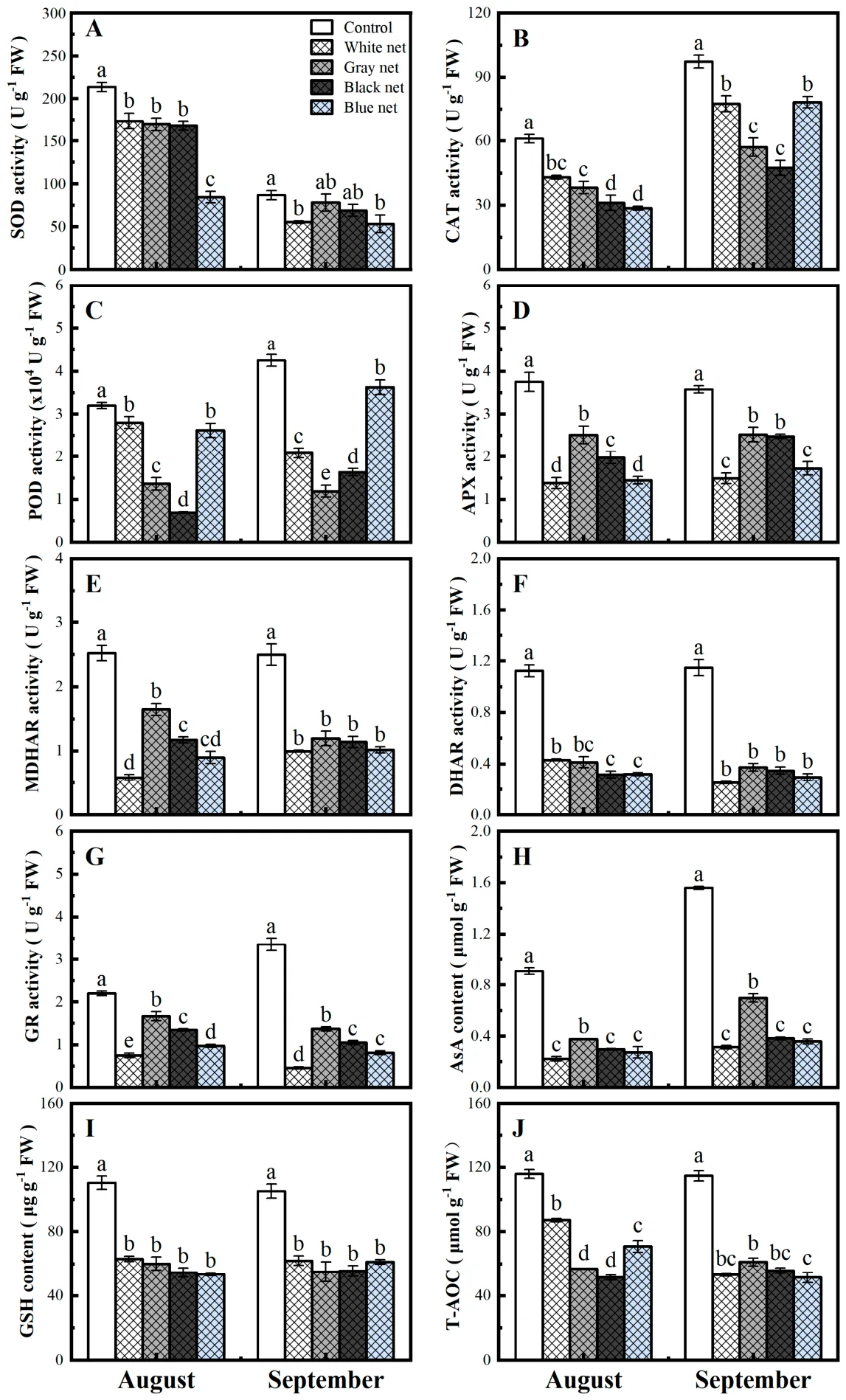
Effects of different colored shade nets on antioxidant enzyme activities, AsA, GSH contents, and T-AOC in ponkan fruit peel in August and September. Superoxide dismutase (SOD) activity (**A**), catalase (CAT) activity (**B**), peroxidase (POD) activity (**C**), ascorbate peroxidase (APX) activity (**D**), monodehydroascorbate reductase (MDHAR) activity (**E**), dehydroascorbate reductase (DHAR) activity (**F**), glutathione reductase (GR) activity (**G**), ascorbic acid (AsA) content (**H**), reduced glutathione (GSH) content (**I**), and total antioxidant capacity (T-AOC) (**J**). Different lowercase letters above bars within the same month indicate significant differences among treatments at *p* < 0.05 by Duncan’s multiple range-test. Data are expressed as means ± standard error (*n* = 3).

As shown in Figure 6A–C, SOD, CAT, and POD activities exhibited distinct responses to shading treatments. In August, SOD activity in peel under all shade-net treatments was significantly lower than that in control plants. SOD activity in plants under the white-net, gray-net, and black-net treatments was significantly higher than that in plants under the blue-net treatment, with no significant differences among the white-net, gray-net, and black-net treatments. In September, SOD activity in plants under the white-net and blue-net treatments was significantly lower than that in control plants, while no significant differences were detected among plants under the four shade-net treatments. For CAT, CAT activity in control plants was significantly higher than that in plants under all shading treatments in both months. In August, no significant difference was detected between plants under the white-net and gray-net treatments; CAT activity in these plants was significantly higher than that in plants under the black-net and blue-net treatments, and there was no significant difference between plants under the black-net and blue-net treatments. In September, CAT activity in plants under the gray-net and black-net treatments was significantly lower than that in control plants and in plants under the white-net and blue-net treatments, and no significant difference in CAT activity was observed between plants under the blue-net and white-net treatments. For POD activity in August, plants under the gray-net and black-net treatments displayed significantly lower values compared with the control, white-net and blue-net treatments, whereas no significant difference was found between the white-net and blue-net groups. In September, POD activity peaked in the control, followed by the blue-net, white-net, black-net, and gray-net treatments; all pairwise comparisons were significantly different. These elevated POD levels in the control indicate stress-induced antioxidant activation in sunburn peel, which was alleviated under shading.

For ascorbate peroxidase (APX) and monodehydroascorbate reductase (MDHAR) (Figure 6D,E), the control was significantly higher than all shading treatments. For APX activity, values under the gray-net and black-net treatments were significantly higher than those of the white-net and blue-net groups in both months. In August, APX activity under the gray-net treatment was significantly greater than that under the black-net treatment. For MDHAR, the gray net was significantly higher than the other three in August, but no differences were found among the shade nets in September. For dehydroascorbate reductase (DHAR) and glutathione reductase (GR) (Figure 6F,G), enzyme activities in plants under all shade-net treatments were significantly lower than those in control plants. DHAR activity in plants under the white-net treatment was significantly higher than that in plants under the black-net and blue-net treatments in August, but no differences were observed in September. GR activity in plants under the gray-net treatment was significantly higher than that in plants under the other treatments in both months.

For ascorbic acid (AsA), reduced glutathione (GSH), and total antioxidant capacity (T-AOC) (Figure 6H–J), all shading treatments significantly reduced AsA and GSH compared with the control. For AsA, the gray net was significantly higher than the other three in both months. For GSH, no differences were found among the shade nets. For T-AOC, T-AOC in control plants was significantly higher than that in plants under all shade nets across both months. In August, T-AOC in plants under the white-net treatment was significantly higher than that in plants under the other three shade-net treatments. In addition, T-AOC in plants under the blue-net treatment was significantly higher than that in plants under the gray-net and black-net treatments, which had the lowest values and did not differ from each other. In September, the T-AOC of the gray net was significantly higher than that of the blue net, while no significant difference was observed between the white and black nets. Such elevated AsA, GSH and T-AOC in the control represented a stress response of sunburned peel, whereas shading reduced this stress load.

### 2.7. Effects of Different Colored Shade Nets on the External and Internal Quality of Ponkan Fruit

**Figure 7:**
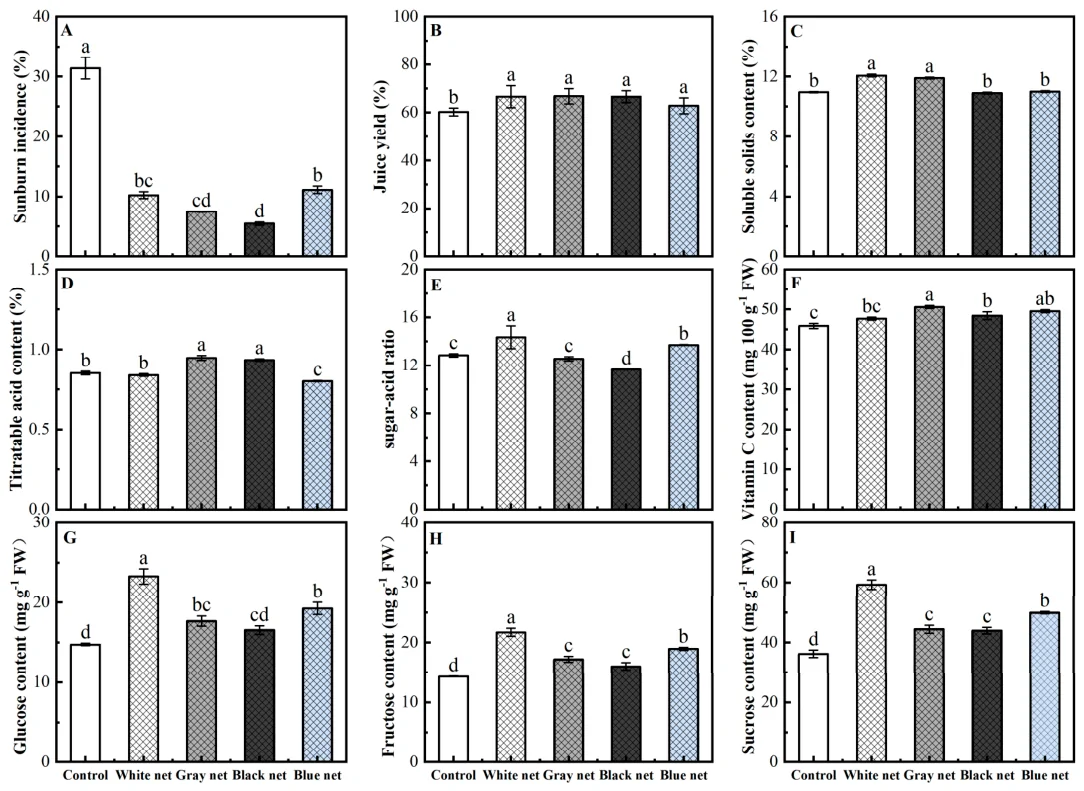
Effects of different colored shade nets on sunburn incidence and fruit quality of ponkan at the ripening stage. Sunburn incidence (**A**), juice yield (**B**), soluble solids content (SSC) (**C**), titratable acid content (TA) (**D**), sugar–acid ratio (**E**), vitamin C content (**F**), glucose content (**G**), fructose content (**H**), and sucrose content (**I**). Different lowercase letters above bars among treatments indicate significant differences among treatments at *p* < 0.05 by Duncan’s multiple range-test. Data are expressed as means ± standard error (*n* = 3).

Figure 7A shows that all colored shade nets effectively reduced fruit sunburn incidence compared with the control. At fruit maturity, no significant differences in fruit external quality (e.g., fruit weight, fruit shape index) were detected across all treatments (Supplementary Table S6). Nevertheless, shading treatments had a significant though treatment-specific impact on fruit internal quality (Figure 7B–F). Most shading treatments elevated juice yield, soluble solids content, and individual sugar levels.

For juice yield, fruit from all shaded treatments had significantly higher juice yield than control fruit; however, no significant differences were detected among the four shade-net treatments. For soluble solids content (SSC), fruit from plants under the white-net and gray-net treatments showed significantly higher SSC than control fruit and fruit from plants under the black-net and blue-net treatments, while fruit from plants under the blue-net and black-net treatments did not differ significantly from control fruit. For titratable acidity (TA), fruit from plants under the gray-net and black-net treatments had significantly higher TA than control fruit, fruit from plants under the blue-net treatment had significantly lower TA than control fruit, and fruit from plants under the white-net treatment showed no significant difference from control fruit (Figure 7B–D). In addition, the sugar–acid ratio was significantly increased only in fruit from plants under the white-net and blue-net treatments, while vitamin C content was significantly enhanced in fruit from plants under the gray-net, black-net, and blue-net treatments (Figure 7E,F).

Under shading, the contents of glucose, fructose, and sucrose in mature fruits were higher than those in the control. For glucose, all shading treatments except the black net showed significantly higher values than the control, while no significant difference was observed between the black net and the control. The ratio of glucose:fructose:sucrose remained approximately 1:1:2 across all treatments. Among the shading treatments, the white and blue nets produced significantly higher fructose and sucrose contents than the gray and black nets, with no significant difference between the gray and black nets. For glucose, fruit from plants under the white-net treatment had significantly higher glucose content than fruit from plants under the gray-net, black-net, and blue-net treatments, and fruit from plants under the blue-net treatment exhibited significantly higher glucose content than fruit from plants under the gray-net and black-net treatments.

## 3. Discussion

### 3.1. Canopy Microclimate Responses and the Scientific Novelty of Colored Shade Nets

A core scientific contribution of this study is the demonstration that different colored shade nets do not simply reduce light intensity, but differentially reshape the canopy microenvironment through selective spectral filtering. Unlike conventional studies focusing solely on the shading rate, our results elucidate the unique contribution of the light quality–thermal environment interaction to the alleviation of photo-thermal stress. For instance, in studies on citrus and other horticultural crops under spectrally selective shade nets, blue nets specifically increased the proportion of 400–500 nm blue light while reducing red-light transmittance, which may benefit stomatal regulation and photomorphogenesis [27,28]. Consistent with those reports, our field data also showed an altered canopy light spectrum under the blue-net treatment, which partially explains the modified physiological responses of ponkan fruit trees. These two moderate-transmittance nets (approx 25–26% transmittance) were significantly higher in light transmittance than the gray and black nets (approx. 13% transmittance), with no significant difference between the white and blue nets. This spectrum-based microenvironmental optimization is the key premise for understanding the differential responses in subsequent leaf photosynthetic performance, antioxidant systems, and fruit quality formation, and also provides a scientific basis for the future precision design of functional covering materials.

Regarding the universality and specificity of shading effects, the present results show both commonalities and differences with previous findings in other species. The cooling effect intensified with increased shading intensity. Fruit surface temperature in plants under the black-net and gray-net treatments was significantly lower than that in plants under the white-net and blue-net treatments, while no significant difference was detected between the black-net and gray-net treatments or between the white-net and blue-net treatments. This trend is consistent with observations in apples, where denser shading lowers fruit surface temperature and illustrates the general physical principle of shading-induced cooling [29]. Drawing on those reported results, our ponkan field data further reveal that light transmittance of shade nets serves as the dominant factor governing fruit surface temperature reduction. However, the regulatory effects of colored shade nets on fruit quality are highly species-specific, leading to divergent results across horticultural crops under similar shading measures. Previous studies have confirmed that appropriate shading benefits fruit coloration and sunburn prevention in apple [30], while our study observed distinct quality responses in ponkan under spectral-selective shading. White- and blue-light-preferred shading environments optimized ponkan sugar–acid balance and sugar accumulation, whereas dense gray and black shading failed to improve fruit quality.

To further interpret the fundamental mechanism underlying such cross-species discrepancies and highlight the innovation of our study, we propose two core hypotheses. First, regional solar radiation intensity and spectral composition vary greatly across planting areas, reshaping the actual microenvironment of shaded crops and inducing divergent physiological responses. Second, different crop species possess distinct physiological response thresholds to light quantity and light quality (e.g., blue/red light ratio and photosynthetic saturation point). These two factors jointly determine the species-specific effects of colored shade nets. Therefore, it is inappropriate to mechanically extend the shading regulation conclusions of other crops to ponkan cultivation, which further demonstrates the practical significance and novelty of our targeted spectral shading regulation research.

### 3.2. Leaf Photosynthetic Acclimation to Colored Shade Nets

Shading significantly altered leaf photosynthetic pigment composition. Total chlorophyll content under all shading treatments was significantly higher than that under the control in September, while in August, the white, gray, and black nets (but not the blue net) showed significantly higher values than the control. This increasing trend with shading intensity represents a typical adaptive strategy to maximize light capture under low-light conditions [14]. The chlorophyll *a*/*b* ratio in leaves of plants under all shade-net treatments was significantly lower than that in control plants under full sunlight in both August and September. Among the shading treatments, the white-net treatment resulted in a higher chlorophyll *a*/*b* ratio than the gray-net and black-net treatments in both months, whereas the blue-net treatment showed no significant difference from the white-net treatment in August. The decrease in the chlorophyll *a*/*b* ratio confirmed that plants broaden their absorption of blue–violet scattered light by increasing chlorophyll *b* content, a common response to shaded environments [31,32]. Total carotenoid content was significantly reduced by all shading treatments compared with the control in both months, with no significant differences among the shade nets, indicating that high light intensity induced the synthesis of photoprotective pigments, whereas moderate shading reduced such protective demands [33,34].

Chlorophyll fluorescence parameters revealed distinct response patterns of photosystem II (PSII) among different treatments. The *F*_v_/*F*_m_ values of leaves in plants under the gray-net and black-net treatments were maintained at approximately 0.77–0.78, indicating the absence of photoinhibition. However, reduced light transmittance under these two shade nets altered light availability and triggered adaptive adjustments in PSII function. In contrast, the control group exhibited a significantly reduced *F*_v_/*F*_m_, falling below 0.80, indicating that PSII suffered photodamage under full-sun conditions. Regarding photosynthetic electron transport, white- and blue-net treatments exhibited significantly higher ETR, *q*P, and ΦPSII values than the gray and black nets, indicating higher photochemical efficiency and effective utilization of absorbed light energy for carbon fixation. These results are consistent with previous findings in apple leaves, where under unshaded conditions at midday, when incident light exceeds the photosynthetic light-saturation point, excess light directly impairs the oxygen-evolving complex. The accumulated ROS further suppress de novo protein synthesis, disrupting the balance between PSII damage and repair and eventually triggering the failure of photoprotective mechanisms rather than their activation [18]. In our ponkan experiment, such severe collapse of photoprotection was largely avoided in plants under shade-net treatments because filtered midday PPFD was kept below this critical high-light threshold; by contrast, non-shaded control ponkan trees experienced comparable high-light stress analogous to the unshaded apple plants reported above. Compared with the white and blue nets, the gray and black nets showed significantly lower *NPQ*, consistent with their reduced ETR and ΦPSII, further indicating impaired photoprotective capacity under these shading conditions.

Photosynthetic gas exchange parameters further support the above differentiation. Plants under the white-net and blue-net treatments achieved significantly higher *P*_n_ than control plants in both months, while plants under the gray-net and black-net treatments showed significantly lower values. Among the shade-net treatments, plants under the white-net treatment had significantly higher *P*_n_ than plants under the blue-net, gray-net, and black-net treatments, with no significant differences among the latter three. This was achieved by maintaining higher *G*_s_ and *C*_i_, indicating that the preservation of photosynthetic potential resulted from the alleviation of midday stomatal limitation. Consistently, Jifon and Syvertsen reported that in citrus leaves under field conditions, moderate shading maintained higher *G*_s_ and *P*_n_ at midday by reducing leaf temperature and vapor pressure deficit, thereby alleviating stomatal restriction to CO_2_ diffusion [35]. In contrast, the significantly reduced *P*_n_ under black and gray nets, accompanied by relatively sufficient *C*_i_, was consistent with non-stomatal limitation, i.e., chronic low-light stress impaired the efficiency of biochemical processes such as electron transport and the Calvin cycle [36]. This differentiated photosynthetic inhibition mechanism—“stomatal limitation under high-light stress vs. metabolic limitation under low-light stress”—represents an important physiological pattern revealed by this study regarding the shading response of ponkan.

### 3.3. Redox Homeostasis and Antioxidant System Regulation by Colored Shade Nets

Shading profoundly influenced reactive ROS metabolic balance in fruit peel by optimizing the field light and thermal microenvironment. Compared with the sunburned control, all shading treatments significantly suppressed the burst of O_2_$\bar{\cdot }$ and H_2_O_2_, and significantly reduced the content of MDA, a product of membrane lipid peroxidation, in both months. In a field experiment on unshaded ‘Golden Delicious’ apple trees under midday high-light stress [18], severe PSII photodamage was triggered when incident light surpassed the photosynthetic saturation point; a similar high-light-driven physiological risk was also observed in non-shaded ponkan control trees in our study.

Regarding the antioxidant system response, the activities of SOD, CAT, and POD in the full-sun control were significantly higher than those in most shading treatments, reflecting stress-induced activation of the enzymatic antioxidant system under severe stress that shading could mitigate [37]. Despite the substantial upregulation of enzyme activities in sunburn peel, they remained insufficient to scavenge the excessively accumulated ROS, ultimately leading to oxidative damage. This “high activity–high damage” pattern has also been reported in studies of high-temperature stress in tomato [38]. The AsA-GSH cycle exhibited fine-tuned regulatory differences among shading treatments. For APX, the gray and black nets showed significantly higher activity than the white and blue nets in both months, except that in August the gray net significantly exceeded the black net. For MDHAR, the gray net was significantly higher than the other three in August, but no differences were found among the shade nets in September. This suggests that even under low-light conditions without significant stress, plants may maintain cellular redox homeostasis through moderate upregulation of the AsA-GSH cycle—a form of adaptive fine-tuning [37,39]. For GR, the gray net exhibited significantly higher activity than the other shading treatments in both months. The significantly higher GR activity in control fruits can be interpreted as a necessary compensatory mechanism to support the continuous operation of the AsA-GSH cycle for regenerating GSH under sustained high-light stress [40], a stress condition relieved by shade-net covering.

The dynamic changes in T-AOC further reinforce the above view. The control exhibited significantly higher T-AOC than all shade nets in both months. In August, the white net was significantly higher than the other three, while in September, the T-AOC of the gray net was significantly higher than that of the blue net, while no significant difference was observed between the white and black nets. In contrast, the stable and relatively low T-AOC levels in shaded fruits reflected a low-load, sustainable steady state of the antioxidant defense system within an optimized microenvironment, fundamentally reducing the long-term oxidative cost to the fruit [39]. By comparison, the gradual decline of T-AOC in sunburn peels after an initial stress-induced increase indicated exhaustion of the antioxidant system, a phenomenon similar to the T-AOC trend observed in ponkan leaves under prolonged drought stress [6].

### 3.4. Fruit Quality Formation via the “Shading–Reillumination” Dynamic Strategy

Shading should not be simplified as a measure to reduce light intensity; instead, it constitutes a dynamic strategy for canopy microclimate optimization. Our data demonstrate that compensatory photosynthesis triggered by reillumination after moderate shading facilitates the late-season accumulation of soluble sugars and vitamin C in ponkan fruit. An optimized microclimate and relieved photo-oxidative stress under appropriate shading lay the physiological foundation for the subsequent carbohydrate accumulation during reillumination. This finding aligns with previous reports showing that short-term shading optimizes citrus fruit quality [41] and agrees with observations in summer-shaded apple orchards, where shading supports the recovery of fruit quality under heat stress [30]. Combined with findings from studies on grape berries indicating that post-shading light recovery activates key genes involved in sugar metabolism and accelerates carbohydrate accumulation [42], we infer that the “shading–reillumination” module is a conserved regulatory mechanism for fruit-quality improvement across perennial fruit crops.

The divergent fruit quality traits observed under various colored shade nets represent the downstream outcomes of spectral reshaping at the canopy scale [12]. Many existing citrus shading investigations rely solely on black shading nets and mainly focus on the incidence of sunburn, while comparative analysis of fruit quality responses to multiple spectral environments remains insufficient, which restricts the establishment of targeted cultivation strategies. Consistent with conventional understanding, all shading treatments improved fruit juice yield compared with the non-shaded control, yet juice yield showed no detectable differences among the colored nets. Light spectrum exerted more pronounced influences on internal quality indicators associated with fruit flavor. White and gray nets significantly raised SSC, whereas blue and black nets caused insignificant SSC variation. The response of titratable acidity was spectrum-dependent: gray and black nets increased TA, while blue nets reduced TA, consequently reshaping fruit sugar–acid balance. The sugar–acid ratio was significantly elevated in fruit from plants under the white-net and blue-net treatments relative to control fruit. This index is regarded as a critical indicator determining fruit sensory quality in apple fruit [43], and this ratio also serves as a key sensory attribute for ponkan. In addition, gray, black and blue nets promoted vitamin C accumulation. All shading treatments enhanced the concentrations of glucose, fructose and sucrose, with the white net generating the highest soluble sugar levels among all tested treatments.

Such differential quality responses highlight distinct application potentials for different covering materials. White and blue nets are advantageous for optimizing sugar–acid balance, while gray and blue nets exhibit unique strengths in boosting vitamin C content. These discrepancies originate from the combined effects of light transmittance and selective spectral filtering. Moderate light availability together with beneficial wavebands (blue light and specific red–orange spectra) sequentially activate light receptors including phytochromes and cryptochromes, and further modulate the transcriptional network governing sugar and organic acid metabolism [44]. Similar light-quality-mediated fruit quality variations have been reported in other horticultural species. However, direct cross-species extrapolation is unreliable, because the outcomes of spectral regulation depend on species-specific physiological traits, local radiation conditions and growth stages. Future work is required to uncover the molecular cascade connecting light signal perception to fruit quality formation in ponkan.

**Figure 8:**
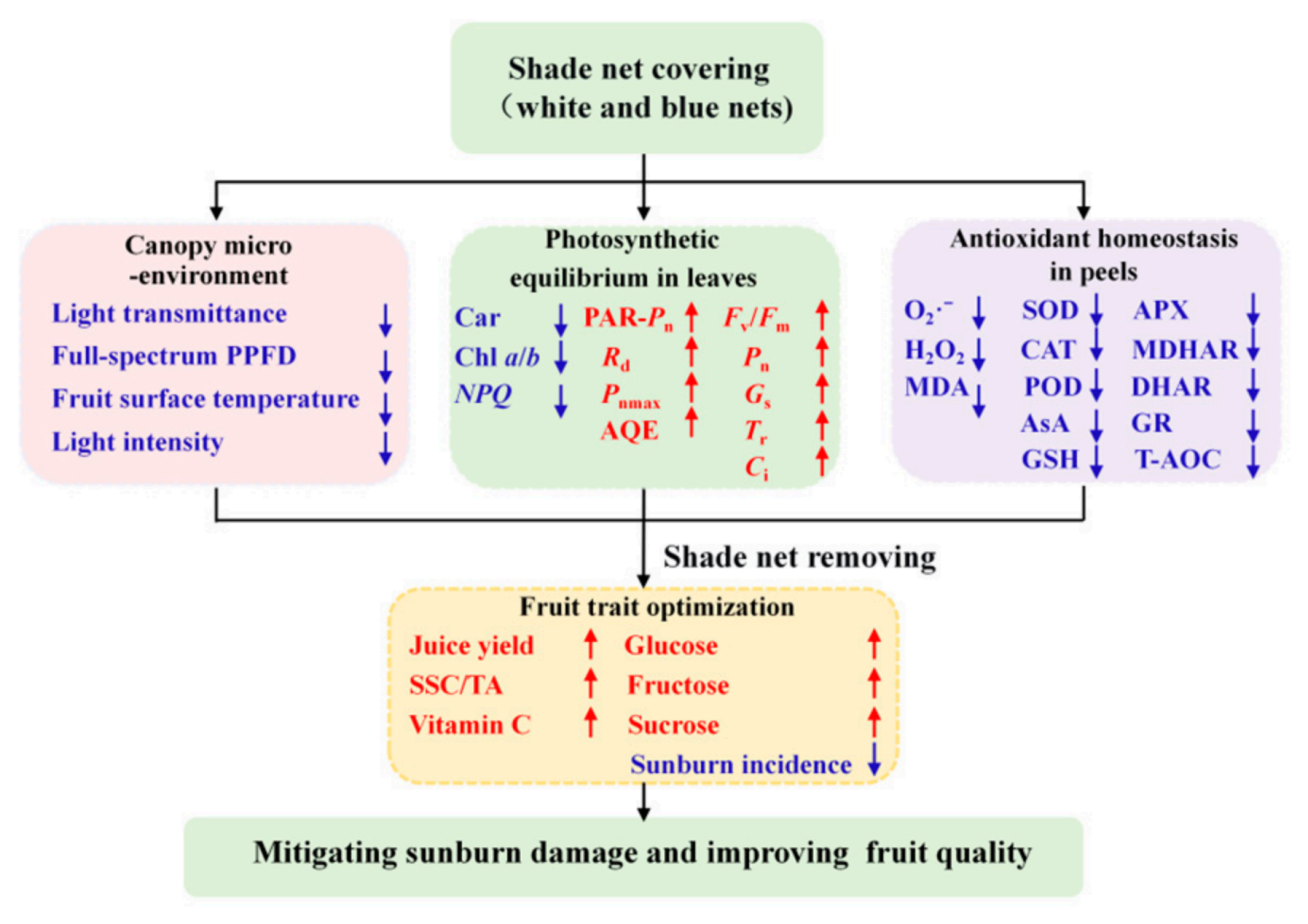
Schematic summary of shared physiological responses measured in this study under white-net and blue-net treatments. In this study, shading was associated with changes in the canopy microenvironment, photosynthetic capacity, antioxidant activity, sunburn incidence, and fruit quality. This schematic is limited to the measured parameters and is not intended to represent a complete regulatory pathway. It only presents the shared responses observed for the two shading-net treatments; treatment-specific divergent effects are described in the main text. Red arrows indicate increases, and blue arrows indicate decreases.

Synthesizing the results across the microclimate, photosynthesis, antioxidant homeostasis, and fruit quality, we found that under the conditions of this study, white-net and blue-net treatments showed favorable effects on the measured parameters. These two treatments were associated with restructuring of canopy microclimates, stable leaf photosynthesis, increased peel antioxidant capacity, alleviated sunburn damage, and improved fruit quality parameters of ponkan. Figure 8 summarizes the shared physiological responses measured in this study that were associated with sunburn mitigation and fruit-quality improvement under white-net and blue-net treatments. This schematic is not intended to represent a complete regulatory pathway, and unmeasured factors may also contribute to the observed effects.

## 4. Materials and Methods

### 4.1. Plant Materials

This experiment was conducted from 15 July to 25 November 2023 at the Tianma Citrus Demonstration Orchard in Yongchun County, Quanzhou City, Fujian Province, China (25°40′ N, 118°29′ E; altitude, 297 m). The study site features a subtropical monsoon climate with distinct seasons and abundant light, heat and precipitation, which is suitable for the year-round growth and development of ponkan. The experimental material was 10-year-old late-maturing ponkan grafted on trifoliate orange (*Poncirus trifoliata*) rootstock. To ensure uniform baseline growth conditions and minimize systematic experimental errors, healthy trees with uniform tree vigor, normal shoot development representative of mature commercial orchards, and stable fruit load, all experimental trees were trained to an open-center system. All experimental trees received consistent irrigation, fertilization, pruning and pest control management throughout the trial period to eliminate interference from cultivation practices.

### 4.2. Shading and Net Removal Regimes, with Leaf Photosynthetic Measurements and Peel Sampling

An IoT-based intelligent agricultural monitoring system (TPSBL-GPRS, TOP Cloud-Agri, Hangzhou, Zhejiang, China) was deployed to record air temperature and photosynthetic photon flux density at the ponkan canopy height at 30 min intervals throughout the shading period. (1) Shading and net removal regimes: shading treatments were imposed from 15 July to 30 September 2023, covering the seasonal high-light and high-temperature stress phase. Five treatments were established: an unshaded blank control and four shading groups covered with white, gray, black, and blue polyethylene shade nets (4 m × 4 m, 6-needle weft-knitted; Shuyang Jinbala E-commerce Co., Ltd., Suqian, Jiangsu, China). Three trees per treatment were selected as biological replicates, with each tree serving as an independent replicate. Each tree bore a stable and substantial crop load (approximately 300–350 fruits per tree), ensuring sufficient tissue availability for repeated non-destructive and destructive sampling. To account for within-tree variability, multiple subsamples (leaves and fruits) were collected from each tree as specified in the respective measurement sections below. This sample size is consistent with previous citrus shading studies [25,29,41]. All shade nets were fully removed on 1 October 2023, when high-light and high-temperature stress subsided and ponkan fruits entered the critical color-transition stage. (2) In situ leaf photosynthetic measurements: on 1 August and 1 September 2023, two dates with the highest incidence of fruit sunburn, in situ determinations of leaf photosynthetic parameters were conducted on canopy leaves from 09:00 to 11:00 using a portable photosynthesis system (CIRAS-4, PP SYSTEMS, Amesbury, MA, USA). (3) Peel tissue sampling: fruit sampling was carried out on the same days as the photosynthetic measurements. Peel tissues were immediately excised, snap-frozen in liquid nitrogen, and stored at −80 °C in darkness for subsequent antioxidant indicator assays. Each biological replicate consisted of peel tissues pooled from 10 fruits, and three biological replicates were prepared for each treatment. All experimental trees were tagged to ensure consistent sampling and physiological monitoring throughout the experimental period. Finally, fruits at commercial maturity (25 November 2023) were harvested for comprehensive quality analysis.

### 4.3. Determination of Light Transmittance and Spectral Composition of Shade Nets

The photosynthetically active radiation (PAR) transmittance and spectral composition of different shade nets were determined prior to the installation of the shade nets over the orchard canopy. All measurements were conducted on a cloudless sunny day (15 July 2023) from 10:00 to 14:00, under consistent weather conditions with sufficient solar irradiance to ensure accurate optical parameter detection. To minimize measurement deviations caused by atmospheric diffuse radiation, all data were collected under unobstructed direct sunlight. A spectrometer (HR-350, HiPoint, Beijing, China) was used to measure PAR intensity both above and below the shade nets at a canopy height of 1.5 m, with the sensor probe positioned horizontally to ensure uniform detection geometry. The same instrument was used to analyze the spectral composition of light transmitted through each shade net over a detection wavelength range of 360–760 nm. For spectral parameter determination, a minimum of three measurement points were recorded per shade net, and the arithmetic mean of all valid replicates was adopted for subsequent statistical analysis.

### 4.4. Monitoring of Fruit Surface Temperature and Light Intensity

Fruit surface temperature and light intensity were monitored on two cloudless sunny days (1 August and 1 September 2023), corresponding to the mid-period of shading treatment. For each treatment, three uniform trees were randomly selected, and five fully sun-exposed fruits per tree were tagged, yielding 15 biological replicates per treatment. Fruit surface temperature was measured using an infrared thermometer (Fluke F62MAX, Fluke Corporation, Everett, WA, USA) with emissivity set to 0.97. The probe was held perpendicular to the fruit equator at a distance of 10 cm, and three stable readings per fruit were averaged for analysis. Light intensity was simultaneously determined with a portable illuminometer (Xima AS-813, Xima Technology, Shenzhen, Guangdong, China) for visible light and a quantum sensor for PAR intensity (μmol m^−2^ s^−1^). Measurements were conducted at 2 h intervals from 08:00 to 16:00.

### 4.5. Measurement and Fitting of Leaf Photosynthetic Light Response Curves

Leaf photosynthetic light response curves were measured on a representative clear, cloudless and windless morning during the peak period of sunburn occurrence, which coincided with the mid-stage of the shading treatment. All leaf samples were collected from the mid-canopy layer (1.5–2.0 m above ground) on the southeastern side of each tree, between 09:00 and 10:00, to minimize diurnal variation and ensure positional consistency across treatments. Measurements were performed with the open gas-path system of a CIRAS-4 portable photosynthesis and fluorescence analyzer (CIRAS-4, PP SYSTEMS, Amesbury, MA, USA). Thirteen gradients of PAR were set as 2000, 1500, 1200, 1000, 800, 600, 500, 400, 300, 200, 100, 50, and 0 μmol m^−2^ s^−1^. Before measurement, tested leaves were acclimated for 20 min under steady pre-illumination conditions (PAR = 1000 μmol m^−2^ s^−1^, CO_2_ concentration = 400 ± 10 μmol mol^−1^, gas flow rate = 200 mmol s^−1^). At each light intensity level, photosynthetic parameters were recorded after 120–300 s of stabilization. Three biological replicates were arranged for each shading treatment, and the average values were adopted for subsequent curve fitting.

The modified rectangular hyperbola model was employed to fit the obtained light response curves [45]. In this model, *P*_n_ represents the net photosynthetic rate, α is apparent initial quantum efficiency, and *R*_d_ indicates the dark respiration rate. Key photosynthetic parameters including AQE, *P*_nmax_, and *R*_d_, together with R^2^, were determined from the model fitting.

### 4.6. Determination of Leaf Chloroplast Pigment Content and Chlorophyll Fluorescence Kinetics Parameters

Leaf chloroplast pigment content was determined using the 95% ethanol extraction method described by Xiong [46]. Briefly, 0.20 g of fresh leaf samples were ground with quartz sand and calcium carbonate in darkness, extracted with 95% ethanol, adjusted to a final volume of 10 mL, and incubated in darkness at room temperature for 30 min. After centrifugation at 5000× *g* for 10 min, the supernatant was collected and diluted to 25 mL with 95% ethanol. Absorbance was measured at 665, 649, and 470 nm using a spectrophotometer (UV-2600, Shimadzu, Japan), with 95% ethanol as the blank. Chlorophyll *a*, chlorophyll *b*, and total carotenoids were quantified at 665 nm, 649 nm, and 470 nm, respectively, and concentrations were calculated according to the equations. Total chlorophyll content was obtained as the sum of chlorophyll *a* and chlorophyll *b*. Pigment contents were calculated and expressed as mg g^−1^ FW. Chlorophyll *a* concentration = 13.95 × A665 − 6.88 × A649; chlorophyll *b* concentration = 24.96 × A649 − 7.32 × A665; carotenoid concentration = (1000 × A470 − 2.05 × Ca − 114.8 × Cb)/245; pigment content = (pigment concentration × total extract volume)/sample mass [33,47].

Chlorophyll fluorescence parameters were measured on fully expanded mature leaves using the integrated fluorescence module of a CIRAS-4 system (PP SYSTEMS, USA). After 30 min of dark adaptation using leaf clips, the minimal fluorescence (*F*_o_) and maximal fluorescence (*F*_m_) were determined, and the maximal photochemical efficiency of PSII was calculated as *F*_v_/*F*_m_ = (*F*_m_ – *F*_o_)/*F*_m_. Under actinic light (2000 μmol m^−2^ s^−1^), steady-state fluorescence (*F*s) and maximal light-adapted fluorescence (*F*_m_’) were recorded. The following parameters were calculated: (1) ETR = ΦPSII × PPFD × 0.5 × 0.84; (2) *q*P = (*F*_m_’ – *F*_s_)/(*F*_m_’ – *F*_o_’); (3) ΦPSII = (*F*_m_’ – *F*_s_)/*F*_m_’; and (4) non-photochemical quenching (*NPQ*) = (*F*_m_ – *F*_m_’)/*F*_m_’. A total of 12 mature, fully expanded leaves per treatment were measured. Specifically, 4 healthy, sun-exposed leaves were selected from the outer middle canopy of each tree (one leaf per cardinal direction), and 3 trees per treatment × 4 leaves per tree = 12 leaves per treatment. All leaf samples were collected from the mid-canopy layer (1.5–2.0 m above ground) on the southeastern side of each tree, between 09:00 and 10:00, to minimize diurnal variation and ensure positional consistency across treatments.

### 4.7. Measurement of Leaf Photosynthetic Gas Exchange Parameters

Leaf gas exchange parameters (*P*_n_, *G*_s_, *T*_r_, and *C*_i_) were measured on two representative clear mornings (1 August and 1 September 2023), corresponding to the middle stage of the shading treatment. Measurements were conducted using the open gas-path system of a CIRAS-4 portable photosynthesis and fluorescence analyzer under fixed conditions (PAR = 1500 μmol m^−2^ s^−1^, CO_2_ = 400 ± 10 μmol mol^−1^, gas flow rate = 200 mmol s^−1^). For each biological replicate, 12 healthy and fully expanded mature leaves of uniform size were selected, and three technical replicate readings were recorded per leaf; all measurements were conducted on fully expanded mature leaves from the mid-canopy layer (1.5–2.0 m above ground) on the southeastern side of each tree. The mean values were used for subsequent analysis.

### 4.8. Determination of O_2_$\bar{\cdot }$, H_2_O_2_ and MDA Content in Peel

The contents of O_2_$\bar{\cdot }$, H_2_O_2_, and MDA in ponkan peel were determined using corresponding assay kits (Solarbio, Beijing, China) according to the manufacturer’s instructions. The results were expressed as μmol g^−1^ FW for O_2_$\bar{\cdot }$ and H_2_O_2_, and as nmol g^−1^ FW for MDA.

### 4.9. Determination of Antioxidant Enzyme Activities in Peel

The activities of SOD, CAT, and POD were quantified using commercial kits from Solarbio (Solarbio, Beijing, China), with absorbance readings taken at 450 nm, 240 nm, and 470 nm, respectively. All enzyme activities were expressed as units per gram of fresh weight (U g^−1^ FW).

### 4.10. Determination of AsA-GSH Cycle Indices in Peel

The AsA and GSH contents were quantified using commercial kits (Solarbio, Beijing, China), with absorbance readings at 265 nm and 412 nm, respectively. The activities of APX, MDHAR, DHAR, and GR were determined using corresponding kits (Solarbio, Beijing, China) according to the manufacturer’s instructions, with absorbance readings at 290 nm, 340 nm, 412 nm, and 340 nm, respectively. The total antioxidant capacity (T-AOC) was measured using a commercial ferric reducing antioxidant power (FRAP) assay kit (Solarbio, Beijing, China), with absorbance at 593 nm. AsA and GSH contents were expressed as μmol g^−1^ FW and μg g^−1^ FW, respectively, whereas enzyme activities and T-AOC were expressed as U g^−1^ FW and μmol g^−1^ FW, respectively.

### 4.11. Determination of Fruit Quality

At fruit commercial maturity (25 November 2023), ten intact fruits of similar size were randomly harvested from the upper canopy of each tree, avoiding the outermost exposed layer and the innermost shaded layer, to ensure representative sampling of marketable fruit. A total of 30 fruits per treatment were collected for quality analyses. For external quality, 30 fruits per treatment were used to measure longitudinal diameter, equatorial diameter, and peel thickness with a Vernier caliper (0.01 mm), and single-fruit fresh weight with an electronic balance (0.01 g); the fruit shape index was then calculated as the ratio of longitudinal diameter to equatorial diameter. For internal quality, juice yield was determined by sequential weighing of whole fruits and the remaining pomace after juice extraction and expressed as a percentage (%). SSC was measured using an Abbe refractometer. TA was determined by acid–base titration with 0.10 mol L^−1^ NaOH and expressed as citric acid equivalent, and the SSC/TA ratio was subsequently calculated. Vitamin C content (mg 100 g^−1^ FW) was measured using the classic 2,6-dichloroindophenol titration method, a standard procedure for fruit samples [48]. Three biological replicates were used per treatment (each pooled from 10 fruits), with three technical repeats per replicate.

### 4.12. Determination of Fruit Soluble Sugar Components by HPLC

For each treatment, ten fruits were randomly selected, and the mixed pulp from these fruits served as one biological replicate, with three replicates in total. The contents of glucose, fructose, and sucrose in ponkan pulp were determined using high-performance liquid chromatography (HPLC, LC-20A, Shimadzu, Japan) following the method of Lu [49] with modifications. Briefly, 0.50–1.00 g of frozen pulp was extracted with 6 mL of distilled water, ultrasonicated for 40 min, and centrifuged at 10,000× *g* for 10 min. The supernatant was filtered through a 0.22 μm aqueous membrane prior to HPLC analysis. Gradient standard solutions of the three sugars were prepared using chromatographic-grade standards and ultrapure water for external calibration. Identification and quantification were achieved by retention time and peak area, respectively. The chromatographic conditions were as follows: mobile phase, acetonitrile: 0.20% ammonia water = 84:16 (V/V); amino column; injection volume, 2.00 μL; flow rate, 0.30 mL min^−1^; column temperature, 35 °C; detector temperature, 80 °C; and run time, 20 min per sample. The three sugars were baseline-separated, and all standard curves exhibited high linearity (R^2^ = 0.9993–0.9999).

### 4.13. Data Statistical Analysis

All raw data were organized and preliminary calculations were performed using Microsoft Excel 2018. Statistical analyses were performed using one-way analysis of variance (ANOVA) independently for each sampling month, followed by Duncan’s multiple range-test for mean comparisons, via SPSS 26.0 (IBM Corp., Armonk, NY, USA). All figures were generated using Origin 2021 (OriginLab Corporation, Northampton, MA, USA). Data are expressed as means ± standard errors (SE). In all figures and tables, different lowercase letters indicate significant differences among treatments within the same month at the *p* < 0.05 level.

## 5. Conclusions

Colored shade nets with differing light transmittance reshape the canopy microclimate of ponkan, triggering divergent physiological and fruit quality responses. All nets lowered fruit surface temperature and alleviated sunburn, but white and blue nets maintained higher leaf photosynthetic capacity and PSII photochemical efficiency, avoiding the chronic low-light stress under gray and black nets. After post-shading light recovery, coordinated photosynthetic acclimation and peel redox homeostasis promoted soluble sugar and vitamin C accumulation in fruit. The white net performed best overall, combining effective sunburn suppression with the highest soluble-sugar content and an improved sugar–acid ratio. The blue net also improved fruit quality, with elevated vitamin C content and sugar–acid ratio; its soluble sugar level was lower than that under the white net but higher than that under the gray and black nets. Gray and black nets provided strong cooling and effective sunburn control but impaired leaf photosynthesis, limiting improvements in key fruit quality traits. Collectively, selective deployment of colored shade nets is a promising strategy to alleviate sunburn and optimize fruit quality in ponkan orchards. This study provides quantitative physiological evidence and practical guidance for orchard light management; the white net is recommended as the preferred option for both sunburn mitigation and superior fruit sensory quality.

## Data Availability

Data are contained within the article. The data presented in this study can be requested from the authors.

## Supplementary Materials

The following supporting information can be downloaded at: https://www.mdpi.com/article/10.3390/plants15182870/s1, Table S1: Spectral distribution of photosynthetic photon flux density (PPFD) under five shading treatments; Table S2: Diurnal variations in fruit surface temperature in August under different colored shade nets; Table S3: Diurnal variations in fruit-surface light intensity in August under different colored shade nets; Table S4: Diurnal variations in fruit surface temperature in September under different colored shade nets; Table S5: Diurnal variations in fruit-surface light intensity in September under different colored shade nets; Table S6: Effects of different colored shade nets on appearance quality of ponkan fruit at the ripening stage.
