# Supplementary material for: Shade Nets Modulate Photosynthetic Balance and Antioxidant Homeostasis to Improve Fruit Quality in Ponkan
Source: Plants (Basel). 2026 Sep 19;15(18):2870. doi: 10.3390/plants15182870 (PMC13610646; doi:10.3390/plants15182870)
Supplement: Supplementary file 1 [file plants-15-02870-s001.zip › plants-4512791-supplementary.pdf]

**Supplementary Material**

**Supplementary Table S1.** Spectral distribution of photosynthetic photon flux density (PPFD) under five shading treatments (corresponding to Figure 1B μmol $m^{-2}$ $s^{-1}$)

| Wavelength (nm) | Control | White net | Gray net | Black net | Blue net |
|---|---|---|---|---|---|
| 400 | 2.95 ± 0.07 a | 0.51 ± 0.02 c | 0.31 ± 0.01 d | 0.39 ± 0.01 cd | 0.83 ± 0.04 b |
| 500 | 6.05 ± 0.32 a | 1.47 ± 0.20 c | 0.72 ± 0.02 d | 0.68 ± 0.03 e | 2.36 ± 0.18 b |
| 600 | 6.84 ± 0.35 a | 1.78 ± 0.15 b | 0.94 ± 0.05 d | 0.87 ± 0.01 e | 1.12 ± 0.14 c |
| 700 | 6.40 ± 0.33 a | 1.82 ± 0.01 bc | 1.02 ± 0.08 d | 0.99 ± 0.04 d | 1.05 ± 0.12 c |

**Note:** Data are expressed as means ± standard error ($n$ = 3). Significant differences among treatments were determined using Duncan's multiple-range test. Different lowercase letters within the same wavelength indicate significant differences at $P < 0.05$. Mean separation is reported via letter grouping.

**Supplementary Table S2.** Diurnal variations in fruit-surface temperature in August under different colored shade nets (corresponding to Figure 1C °C)

| Time | Control | White net | Gray net | Black net | Blue net |
|---|---|---|---|---|---|
| 8:00 | 41.35 ± 0.58 a | 32.07 ± 0.48 b | 32.17 ± 1.82 b | 31.67 ± 1.42 b | 31.72 ± 0.72 b |
| 10:00 | 44.67 ± 0.88 a | 35.70 ± 1.25 b | 36.02 ± 0.83 b | 35.70 ± 1.05 b | 35.27 ± 1.04 b |
| 12:00 | 48.18 ± 1.04 a | 38.50 ± 2.15 b | 36.65 ± 1.24 b | 36.63 ± 1.82 b | 37.90 ± 0.42 b |
| 14:00 | 44.22 ± 1.69 a | 35.31 ± 0.48 b | 36.23 ± 0.65 b | 35.49 ± 0.99 b | 36.26 ± 0.85 b |
| 16:00 | 42.58 ± 0.93 a | 33.45 ± 0.51 b | 35.19 ± 1.51 b | 34.05 ± 1.08 b | 35.14 ± 0.88 b |

**Note:** Data are expressed as means ± standard error ($n$ = 3). Significant differences among treatments were determined using Duncan's multiple-range test. Different lowercase letters within the same time point indicate significant differences at $P < 0.05$. Mean separation is reported via letter grouping.

**Supplementary Table S3.** Diurnal variations in fruit-surface light intensity in August under different colored shade nets (corresponding to Figure 1D μmol $m^{-2}$ $s^{-1}$)

| Time | Control | White net | Gray net | Black net | Blue net |
|---|---|---|---|---|---|
| 8:00 | 554.40 ± 34.99 a | 233.86 ± 24.23 b | 89.56 ± 12.84 c | 56.61 ± 11.56 c | 114.50 ± 19.99 c |
| 10:00 | 1723.76 ± 47.21 a | 440.53 ± 34.23 b | 117.97 ± 17.63 d | 117.10 ± 16.00 d | 330.74 ± 29.99 c |
| 12:00 | 1955.00 ± 49.52 a | 467.35 ± 38.00 b | 130.45 ± 19.99 c | 128.71 ± 18.46 c | 361.09 ± 34.50 b |
| 14:00 | 1551.40 ± 44.60 a | 401.24 ± 32.23 b | 134.58 ± 16.00 c | 121.26 ± 14.99 c | 354.14 ± 27.06 b |
| 16:00 | 559.14 ± 37.99 a | 275.09 ± 27.12 b | 55.31 ± 11.67 d | 53.61 ± 13.00 d | 189.35 ± 21.05 c |

**Note:** Data are expressed as means ± standard error ($n$ = 3). Significant differences among treatments were determined using Duncan's multiple-range test. Different lowercase letters within the same time point indicate significant differences at $P < 0.05$. Mean separation is reported via letter grouping.

**Supplementary Table S4.** Diurnal variations in fruit-surface temperature in September under different colored shade nets (corresponding to Figure 1E, °C)

| Time | Control | White net | Gray net | Black net | Blue net |
|---|---|---|---|---|---|
| 8:00 | 33.10 ± 0.68 a | 32.00 ± 0.96 b | 28.60 ± 0.53 b | 30.30 ± 1.39 b | 30.30± 0.99 b |
| 10:00 | 38.69 ± 0.23 a | 33.30 ± 0.84 b | 33.10 ± 1.71 b | 33.30 ± 1.55 b | 32.90 ± 1.23 b |
| 12:00 | 47.20 ± 0.69 a | 37.40 ± 0.38 b | 36.29 ± 0.89 b | 35.90 ± 0.71 b | 36.88 ± 1.25 b |
| 14:00 | 41.60 ± 1.05 a | 35.40 ± 1.14 b | 34.12 ± 0.59 b | 33.80 ± 1.20 b | 34.52 ± 0.61 b |
| 16:00 | 39.20± 1.57 a | 33.20 ± 1.04 b | 32.50 ± 0.99 b | 31.35 ± 0.84 b | 32.60 ± 1.15 b |

**Note:** Data are expressed as means ± standard error ($n$ = 3). Significant differences among treatments were determined using Duncan's multiple-range test. Different lowercase letters within the same time point indicate significant differences at $P < 0.05$. Mean separation is reported via letter grouping.

**Supplementary Table S5.** Diurnal variations in fruit-surface light intensity in September under different colored shade nets (corresponding to Figure 1F, μmol $m^{-2}$ $s^{-1}$)

| Time | Control | White net | Gray net | Black net | Blue net |
|---|---|---|---|---|---|
| 8:00 | 544.59 ± 45.92 a | 172.01 ± 14.67 b | 56.61 ± 3.71 c | 45.33 ± 6.35 c | 128.00 ± 4.62 b |
| 10:00 | 1105.10 ± 36.91 a | 314.60 ± 26.58 b | 117.10 ± 6.74 c | 58.41 ± 1.55 c | 266.30 ± 3.14 b |
| 12:00 | 1777.30 ± 22.21 a | 422.80 ± 19.21 b | 124.45 ± 10.16 c | 101.43 ±7.98 c | 367.10 ± 13.75 b |
| 14:00 | 1279.40 ± 18.88 a | 378.60 ± 12.61 b | 124.58 ± 6.26 d | 103.55 ± 15.92 d | 251.85 ± 22.08 c |
| 16:00 | 513.10 ± 15.61 a | 175.67 ± 4.12 b | 55.31 ± 4.18 c | 54.68 ± 6.63 c | 155.37 ± 15.98 b |

**Note:** Data are expressed as means ± standard error ($n$ = 3). Significant differences among treatments were determined using Duncan's multiple-range test. Different lowercase letters within the same time point indicate significant differences at $P$ < 0.05. Mean separation is reported via letter grouping.

**Supplementary Table S6.** Effects of different colored shade nets on appearance quality of ponkan fruit at the ripening stage

| Treatments | Transverse Diameter / mm | Longitudinal Diameter / mm | Fruit weight / g | Fruit shape index |
|---|---|---|---|---|
| Control | 70.59 ± 5.90 | 57.37 ± 1.07 | 176.40 ± 6.74 | 0.75 ± 0.01 |
| White net | 73.57 ± 0.61 | 54.81 ± 0.62 | 173.75 ± 10.2 | 0.74 ± 0.02 |
| Gray net | 75.51 ± 2.02 | 57.15 ± 1.48 | 164.02 ± 5.01 | 0.75 ± 0.01 |
| Black net | 71.48 ± 0.63 | 55.71 ± 1.14 | 154.85 ± 3.86 | 0.78 ± 0.02 |
| Blue net | 75.92 ± 1.58 | 56.88 ± 0.55 | 168.94 ± 8.34 | 0.75 ± 0.01 |

**Note:** Data are expressed as means ± standard error ($n$ = 3). Different lowercase letters within the same indicator indicate significant differences among treatments at $P$ < 0.05 according to Duncan's multiple-range test.
